# Flowable composite as an alternative to adhesive resin cement in bonding hybrid CAD/CAM materials: in-vitro study of micro-shear bond strength

**DOI:** 10.1038/s41405-024-00251-2

**Published:** 2024-08-20

**Authors:** Eman Ezzat Youssef Hassanien, Zeinab Omar Tolba

**Affiliations:** 1 Fixed Prosthodontics Department, Faculty of Dentistry, Cairo University, Cairo, Egypt; 2 Conservative Dentistry Department, Faculty of Dentistry, Cairo University, Cairo, Egypt

**Keywords:** Fixed prosthodontics, Bonded restorations

## Abstract

**Objective:**

To assess the micro-shear bond strength of light-cured adhesive resin cement compared to flowable composite to hybrid CAD/CAM ceramics.

**Materials and methods:**

Rectangular discs were obtained from polymer-infiltrated (Vita Enamic; VE) and nano-hybrid resin-matrix (Voco Grandio; GR) ceramic blocks and randomly divided according to the luting agent; light-cured resin cement (Calibra Veneer; C) and flowable composite (Neo Spectra ST flow; F), resulting in four subgroups; VE-C, VE-F, GR-C and GR-F. Substrates received micro-cylinders of the tested luting agents (*n* = 16). After water storage, specimens were tested for micro-shear bond strength (µSBS) using a universal testing machine at 0.5 mm/min cross-head speed until failure and failure modes were determined. After testing for normality, quantitative data were expressed as mean and standard deviation, whereas, qualitative data were expressed as percentages. Quantitative data were statistically analysed using Student *t* test at a level of significance (*P* ≤ 0.05).

**Results:**

Group GR-F showed the highest µSBS, followed by VE-C, VE-F and GR-C respectively, although statistically insignificant. All groups showed mixed and adhesive failure modes, where VE-F and GR-C showed the highest mixed failures followed by GR-C and VE-C respectively.

**Conclusions:**

After short-term aging, flowable composite and light-cured resin cement showed high comparable bond strength when cementing VE and GR.

## Introduction

Conservative restorations; such as laminate veneers and occlusal veneers, became highly desired nowadays. Such restorations provided maximum tooth preservation and high patients’ satisfaction [1]. Wide variety of new CAD/CAM materials has been developed to be used in fabricating such restorations aiming to attain high aesthetics while maintaining optimum mechanical properties.

Conventional ceramics offer excellent aesthetics, colour stability, biocompatibility and serviceability [2, 3]. However, their brittleness and the possibility of wearing the opposing dentition during mastication made their use challenging [2, 3]. On the other hand, composite resin blocks offered good machinability and low wear to the opposing dentition, however, they suffered from increased material wear with loss of surface polish and colour instability [2, 3].

Recently, hybrid or resin-matrix ceramics were introduced to combine the advantages of both ceramics and polymers, aiming to imitate the mechanical behaviour of the natural teeth, while maintaining high aesthetics [2]. Vita Enamic (VE); a polymer-infiltrated ceramic-network material, possessed a unique structure of two three-dimensional interpenetrating networks comprising a dominant ceramic network (86 wt%) and a polymer network (14 wt%) [4, 5]. Voco Grandio (GR), a nano-hybrid resin-matrix ceramic, also consisted of predominant inorganic fillers (86 wt%) in addition to the organic portion [6].

Both VE and GR offered reduced brittleness [2], high resilience, flexibility and fatigue resistance [6] to better withstand the exerted masticatory forces compared to conventional ceramics. They also offered good bond strength and wear resistance with low abrasion to the opposing teeth [2, 6]. Being supplied as CAD/CAM blocks, they gained the advantages of the digital workflow, which allowed the production of restorations with high precision and accuracy in short time compared with the conventional approach [3, 7]. In addition, compared to conventional ceramic materials, they offered fast machinability without the need of additional firing, glazing or crystalizing procedures [2, 6]. Furthermore, they allowed easy intra-oral reparability and polishing [6], which aided in time-saving and ease of construction.

However, long-term survival of conservative indirect restorations does not depend solely on the restorative material used. It depends greatly on establishing an efficient bond between such restorative material and the prepared tooth structure [8]. Poor bonding might decrease the restoration fracture strength, retention and increase the risk of micro-leakage [6, 9].

For many years, light-cured adhesive resin cement was the material of choice when cementing conservative indirect aesthetic restorations, owing to their high mechanical properties, low solubility, high colour stability and controllable working time [10–14]. However, their use possessed some challenges, where improper handling might cause premature curing with or without the presence of excess cement residing in undesirable areas and difficult to remove. Additionally, their relatively low inorganic filler content, which contributes to their high flow, could increase the volumetric polymerization shrinkage and result in a thermal expansion coefficient higher than that of enamel and dentin, which can subsequently result in interface failure; exposing the cement to the oral environment and compromising the restoration longevity and aesthetics [14, 15].

On the other hand, dual-cured resin cements possessed higher mechanical properties; such as flexural strength, hardness and elastic modulus, when compared to light-cured resin cements [10, 13]. They also showed a higher degree of conversion [10, 13] due to their dual activation modes, with better physicochemical properties [10]. However, these cements suffered from a shorter working time and colour instability [10, 13], which possessed a problem in aesthetic restorations. Hence, alternative materials were investigated.

Some researchers speculated that using composite resins; with higher inorganic filler content [10, 14, 16] and lower initiators concentration [14], might be advantageous in all ceramic restorations cementation, because they possessed higher colour stability and mechanical wear resistance [14, 16] compared to dual-cured resin cements. However, such increase in the filler content increased the material viscosity, which represented a challenge during restoration seating and caused a thick cement line at the interface [16].

Hence, attempts were made to reduce such viscosity by preheating. It was believed that preheating can improve the material flow [16]; allowing better restoration adaptation [10], thin cement line [16], lower defects at the margins [10], and increased degree of conversion with better physical and mechanical properties at low cost [10].

However, preheating is technique sensitive [14] and added an additional step to the clinical procedure [14], which is considered a limitation. Additionally, it was found that the preheating effect varied according to the resin composite type [10, 15, 16], composition [10], filler content and size [10, 16], and photo-initiator system [10]. Some researchers found that preheated nano-hybrid resin formed a thicker film than that formed by preheated micro-hybrid resin [16]. Preheating temperature and time [16] is also an affecting factor; where different composite resins take different times to reach a stable temperature. Additionally, prolonged heating in ovens or warmers can cause some low molecular weight components of the photo-initiator system to volatilize [16]. Although increasing the degree of conversion by preheating can reduce the light-polymerization time, ideal light-polymerizing time or intensity has not yet been determined [16]. Furthermore, cementation with preheated composite resins requires higher pressure during the restoration placement, thus, in case of very thin veneers, the possibility of crack and fracture increases [10]. Hence, using materials that possess high filler content with high flow and do not require additional preheating step; such as flowable composites, might be beneficial.

Flowable composites were first introduced as conservative restorative materials, cavity liners, pits and fissure sealants [17]. However, their use has expanded lately to involve other applications including orthodontic bracket and retainer bonding, splinting fractured teeth, repairing provisional restorations and recently veneers cementation [13, 17, 18]. Flowable composite, is an adhesive material, with particle size similar to that of the hybrid composites but possesses lower viscosity while maintaining excellent handling properties [13, 17]. Their remarkable effective penetration of surface irregularities with adequate surface wetting ability [13, 18, 19], radio-opacity, shade variety and improved cost benefit compared to resin cements made them a valuable alternative to adhesive resin cement in luting conservative restorations [13, 18]. Both light-cured resin cements and flowable composites contained low concentration of tertiary amines offering greater colour stability compared to dual-cued resin cement [8].

Compared to resin cements, the advantages of flowable composites lie in combining higher filler content with low viscosity and clinical procedures simplicity at lower cost. The higher filler content is beneficial in improving their physical properties [20], which might aid in indirect conservative restoration longevity. Their viscosity facilitated pre-polymerization clean-up without the need for partial or tack polymerization employed in resin cements [21] in addition to allowing low film thickness and facilitating thin restoration seating [15] with minimum pressure. Furthermore, their high flexibility allowed them to be less prone to displacement in high-stress areas [19]. It was also believed that their use can prevent bubble incorporation or entrapment [21], which might occur when using resin cements that employ mixing two components [14].

Several studies were conducted to test the efficiency of using flowable composite as a luting agent compared to resin cements, regarding different parameters such as colour stability and opacity [13], micro-tensile bond strength [22], compressive strength [23], radiant exitance, degree of conversion [15, 24], shrinkage strain, polymerization stress, elastic modulus [15], florescence [20] and shear bond strength to feldspathic porcelain [21] and lithium disilicate [18]. However, the difference between their bondability to polymer-infiltrated ceramic network and nano-hybrid resin-matrix ceramic is still unclear.

To test bonding in-vitro, several tests were commonly used, including shear, micro-shear, tensile and micro-tensile bond strength tests. Micro-shear bond strength test (μSBS) offered simple testing protocol, with good control on the bonded area by using standardized micro-tubes of known diameter [25–27].

For better simulation of intra-oral conditions, aging is usually employed in in-vitro studies. Water storage, a popular aging method, offered an easy, simple, low-cost method with a well-known effect on degrading bonded interfaces [28]. The high molar concentration of water and its small molecular size allowed its penetration in the small spaces between the polymer chains or functional groups, which deteriorated the polymer thermal stability causing its plasticization and subsequently hydrolytic degradation of resin luting agents [29]. Hence, it was employed in many in-vitro studies that tested bonding strength.

To the best knowledge of the authors, limited data are available comparing flowable composite bonding performance to that of adhesive resin cement especially when using advanced ceramic materials. Thus, the present study aimed to evaluate the micro-shear bond strength of flowable composite to recent CAD/CAM materials (VE and GR) in comparison to adhesive resin cement after aging. The first tested null hypothesis was that there would be no statistically significant effect of the luting material on micro-shear bond strength of VE and GR materials, while the second null hypothesis was that, regardless of the luting material used, there would be no statistically significant difference between the bond strength of either VE or GR.

## Materials and methods

### The study design, ethical approval, and sample size calculation

The present study is an in-vitro research performed in a randomized and blinded manner. The study received ethical approval from the Ethics Committee of Scientific Research—Faculty of Dentistry—Cairo University.

Prior to conducting the study, sample size calculation was performed using PS: Power and Sample Size Calculations software (version 3.2.1, Vanderbilt University), adopting 0.05 alpha level of significance and a power of 80% rendering a total of 64 samples.

### Materials

**Fig. 1:**
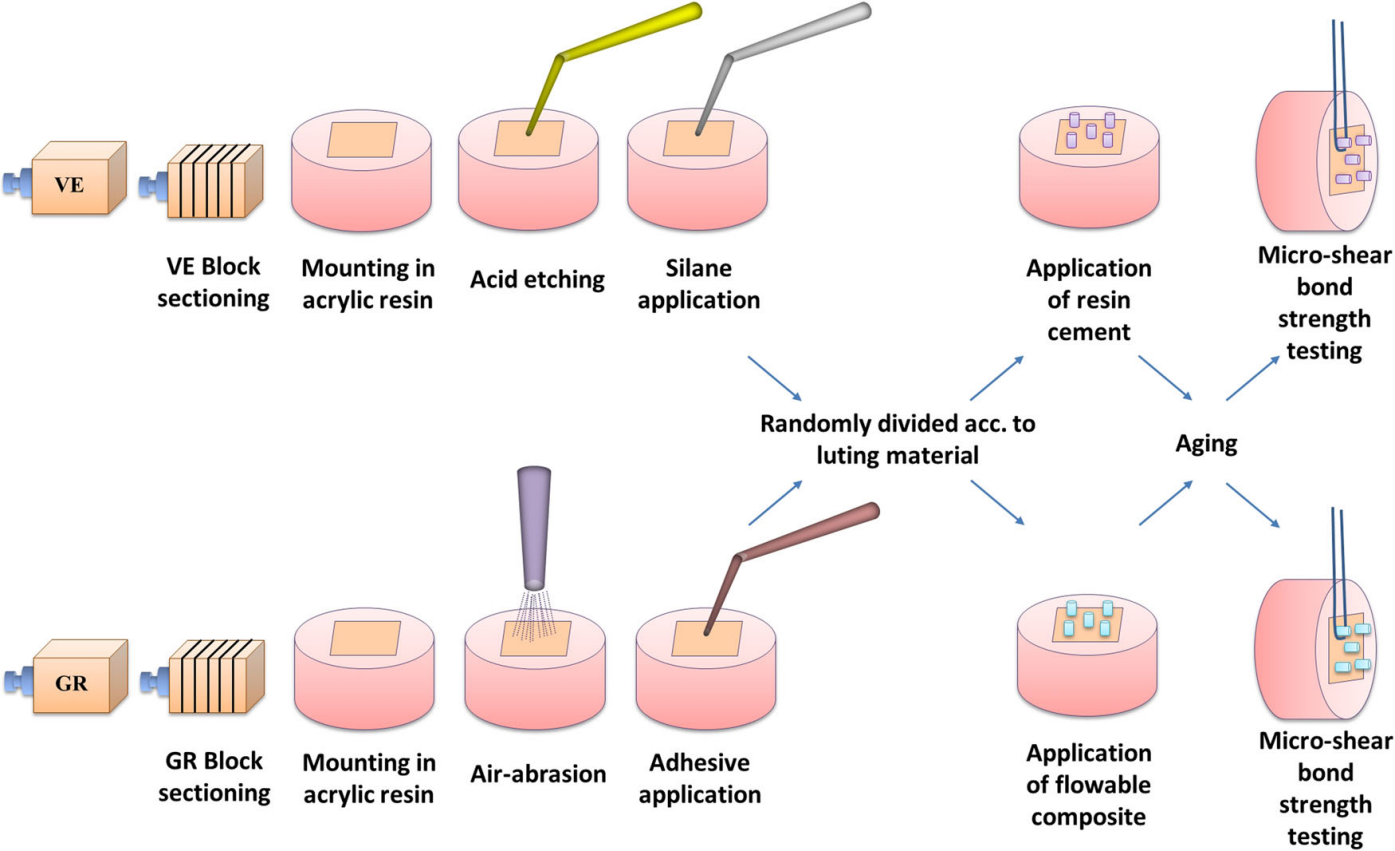
Schematic representation of the specimens’ preparation and testing. (VE VITA Enamic, GR Voco Grandio).

The materials used in the present study, their description, composition, manufacturer and batch number are listed in Table 1; whereas, the study experimental design is shown in Fig. 1.

**Table 1:** The name, description, composition, manufacturer and batch number of the materials used in the present study.

Material name	Description	Composition	Manufacturer	Batch no.
VITA Enamic (2M2-T, EM-14)	Polymer-infiltrated ceramic network CAD/CAM blocks	Inorganic ceramic part (86 wt%): 58–63% silicon dioxide, 20–23% aluminum oxide, 9–11% sodium oxide, 4–6% potassium oxide, 0.5–2% boron trioxide, <1% zirconium dioxide, <1% calcium oxide Organic polymer part: UDMA, TEGDMA	VITA Zahnfabrik, Bad Säckingen, Germany	74550
Voco Grandio blocs (14L, A2, LT)	Nano-hybrid resin-matrix ceramic CAD/CAM blocks	Inorganic ceramic part (86 wt%): nano-hybrid fillers Organic polymer part: UDMA + DMA	VOCO GmbH, Cuxhaven, Germany	2313294
Calibra Veneer Esthetic Resin Cement (Translucent)	Light-cured resin cement	Dimethacrylate resins, Camphorquinone (CQ), Photoinitiator, Stabilizers, Glass Fillers, Fumed silica, Titanium Dioxide, Pigments.	Dentsply, Sirona, Germany	00086060
Neo Spectra ST flow (A2)	Light-cured flowable composite material	Filler matrix (62.5 wt%): Barium-aluminum-borosilicate glass, ytterbium fluoride, iron oxide pigments, titanium oxide pigments Resin matrix: Urethane modified Bis-GMA-adduct, Bis-EMA, Camphorquinone (CQ) Photoinitiator, stabilizers, diluents, pigments	Dentsply, Germany	2007000386
Ceramic Bond	Coupling agent	Organic acid, 3-methacryl oxypropyltrimethoxysilane and acetone	VOCO GmbH, Cuxhaven, Germany	2141131

*wt.* weight, *UDMA* urethane dimethacrylate, *TEGDMA* triethylenglycol dimethacrylate, *DMA* dimethacrylate, *Bis-EMA* ethoxylated bisphenol-A-diglycidyl methacrylate, *Bis-GMA* bisphenol-A-glycidyl methacrylate.

### Substrates preparation

Two groups of rectangular-shaped discs (12 × 14 × 2 mm) were obtained from VITA Enamic (VE; VITA Zahnfabrik, Bad Säckingen, Germany) and Voco Grandio (GR; VOCO GmbH, Cuxhaven, Germany) blocks using linear precision cutting machine (IsoMet 4000, Buehler, USA) at low-speed of 2500 rpm under copious water to avoid heat generation [17, 30, 31].

All discs were inspected for any evident defects and checked using a precise digital caliper (Digital Vernier Caliper IP54, USA) to verify their thickness. Defect-free discs were individually embedded in auto-polymerizing acrylic resin (Acrostone, Acrostone Co Ltd, Egypt) block to facilitate their handling and testing. All substrates were ultrasonically cleaned (CODYSON, CD-4820, China) in distilled water [6] for 10 min and air-dried [30, 32] to eliminate any residual debris. Each substrate was then placed in a small numbered sealed plastic bag to protect its bonded surface from scratching or contamination and to help in randomization and blinding.

### Substrates randomization and subgrouping

Each group of substrates was randomly divided into two subgroups according to the luting material tested (*n* = 16 per subgroup); VE-C: VE discs receiving light-cured resin cement, VE-F: VE discs receiving flowable composite, GR-C: GR discs receiving light-cured resin cement, and GR-F: GR discs receiving flowable composite. Randomization of substrates was performed using randomized sequence lists generated using computer software (www.random.org) to ensure bias elimination and guarantee results reliability.

### Substrates surface treatment

#### For GR substrates

**Fig. 2:**
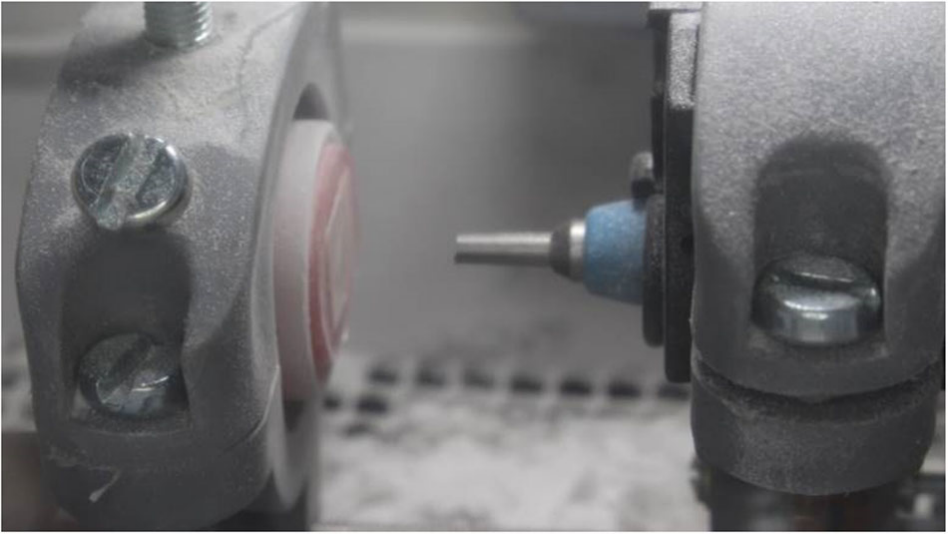
GR substrates being air-abraded aided by the custom-made holding device.

The discs’ exposed surface was air-abraded (Basic eco Fine sandblasting unit, Renfert GmbH, Germany) using 50 μm Al_2_O_3_ particle at 1.5 bar pressure as recommended by the manufacturer. To standardize the distance between the air-abrading machine nozzle and the substrates at 10 mm, a custom-made holding device was constructed (Fig. 2). The device comprised housing for the air-abrading device hand-piece and another for the substrate. Each housing comprised tightening screws to allow proper adjustments. After air-abrasion, Gr substrates were cleaned using steam cleaner (LIZHONG, China) and dried with oil-free air to remove remnants of air-abrasion particles. A bonding agent (Ceramic bond, VOCO GmbH, Germany) was then applied to the air-abraded surface by disposable micro-brush for 60 s and left to dry according to the manufacturer’s recommendations.

#### For VE substrates

The discs’ exposed surface was etched using 9% buffered hydrofluoric acid (Ultradent Porcelain Etch, Ultradent Products Inc, USA) for 60 s then thoroughly rinsed with water for 20 s and dried for 10 s with oil-free air. Silane coupling agent (Ultradent Silane, Ultradent Products Inc, USA) was then applied to the etched surface with disposable micro-brush and let dry for 60 s.

### Preparation of luting agent micro-cylinders

Transparent polyvinyl [6] tubes of 1.44 mm internal diameter, were cut with the help of an endo-ruler and sharp scalpel (blade #15, Wuxi Xinda Medical Device Co Ltd., China) to obtain equal small micro-tubes of 2-mm height [33]. The cut micro-tubes were checked meticulously and any micro-tube that showed irregular edges or was cut at an angle other than 90° was discarded and replaced.

Each micro-tube was then placed perpendicular to the bonded surface, held in place with a dental tweezer, and carefully filled with the tested luting material; light-cured resin cement (Calibra Veneer Esthetic Resin Cement, Dentsply, Sirona, Germany) and light-cured flowable composite (Neo Spectra ST flow, Dentsply, Sirona, Germany) by a single skilled operator to ensure standardization. Light-curing (3M ESPE Elipar DeepCure-L, 3M ESPE, St Paul, MN, USA) was performed according to the manufacturer’s instructions for 20 s at each side of the tubes for both materials after excess material removal [34, 35].

After complete setting, the micro-tubes were carefully removed after being sectioned with a sharp scalpel (blade #15) [6, 34] and the luting material micro-cylinders were checked visually for interface integrity free from air bubbles, gaps, defects or excess material [31].

### Aging and micro-shear bond strength (μ-SBS) test

All specimens in both groups were stored in distilled water at room temperature [36, 37] for 21 days to simulate short-term intra-oral aging. A universal testing machine (Model 3345; Instron Industrial Products, Norwood, MA, USA) equipped with a load cell of 5 KN was used to test the micro-shear bond strength. The test was performed by a single assessor, who was blinded to the tested materials with the help of the numbers [38] that was given to the specimens earlier. The acrylic resin base was secured to the machine’s lower fixed compartment and each bonded micro-cylinder was subjected to a shearing load at a crosshead speed of 0.5 mm/min [6, 39, 40] until failure using orthodontic-wire loop method, where a thin loop-shaped stainless steel orthodontic wire (0.014-inch diameter) was wrapped around each micro-cylinder in contact with the ceramic-resin interface [17, 34]. The force required for debonding was recorded in Newton (N) [6, 17, 34] by a computer software (Instron® Bluehill Lite Software). Data were then calculated in mega-pascals (MPa) by the equation:

$${{{\bf{R}}}}={{{\bf{F}}}}/{{{\bf{A}}}}$$

Where; *R*: μ-shear bond strength (MPa), *F*: load to failure (N), *A*: circular interface area (mm^2^) calculated by the equation *A* = *πr*^2^, where *π* = 3.14 and r: internal radius of micro-cylinder (0.72 mm) [29, 33] resulting in ≈1.63 mm^2^ area.

### Failure mode/pattern analysis

The debonded surfaces were examined for failure mode determination using a stereomicroscope [3, 6, 14, 32, 41–44] (Leica MZ6, Leica Microsystems, Switzerland) at 20× magnification. The failure modes were classified into; adhesive: at the interface, cohesive: within the luting material or the substrate and mixed: involving both adhesive and cohesive failures [6, 17, 34, 45]. Representative specimens of different failure patterns were further examined using a high-resolution scanning electron microscope [41, 42, 44] (QUANTA FEG250, FEI Company, Netherlands) at 20 kV and 120× magnification. Both the operator of the stereomicroscope and the operator of the SEM were blinded to the tested groups using the number codes as previously mentioned.

### Statistical analysis

Data were statistically analysed by an expert statistician, who was also blinded to the tested groups, using statistical software (IBM SPSS Statistics for Windows, Version 25.0., IBM Corp, USA). After checking for normality using Kolmogorov–Smirnov test, quantitative data were found to be parametric and were expressed as mean and standard deviation (SD) then compared using Student *t* test. On the other hand, qualitative variables were expressed as percentages. The level of significance was set at *P* value ≤ 0.05 for the present study.

## Results

**Fig. 3:**
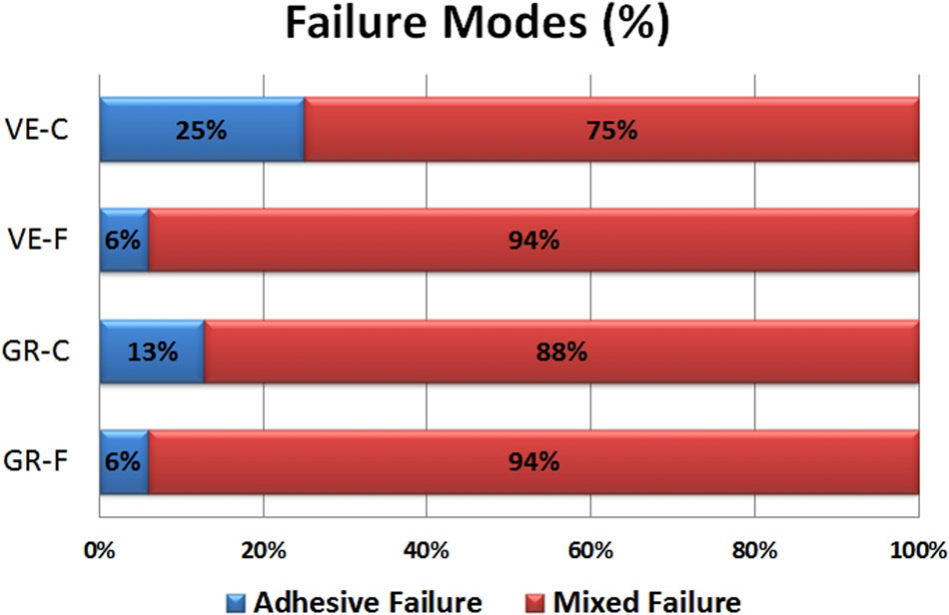
The failure modes of the tested specimens.

**Fig. 4:**
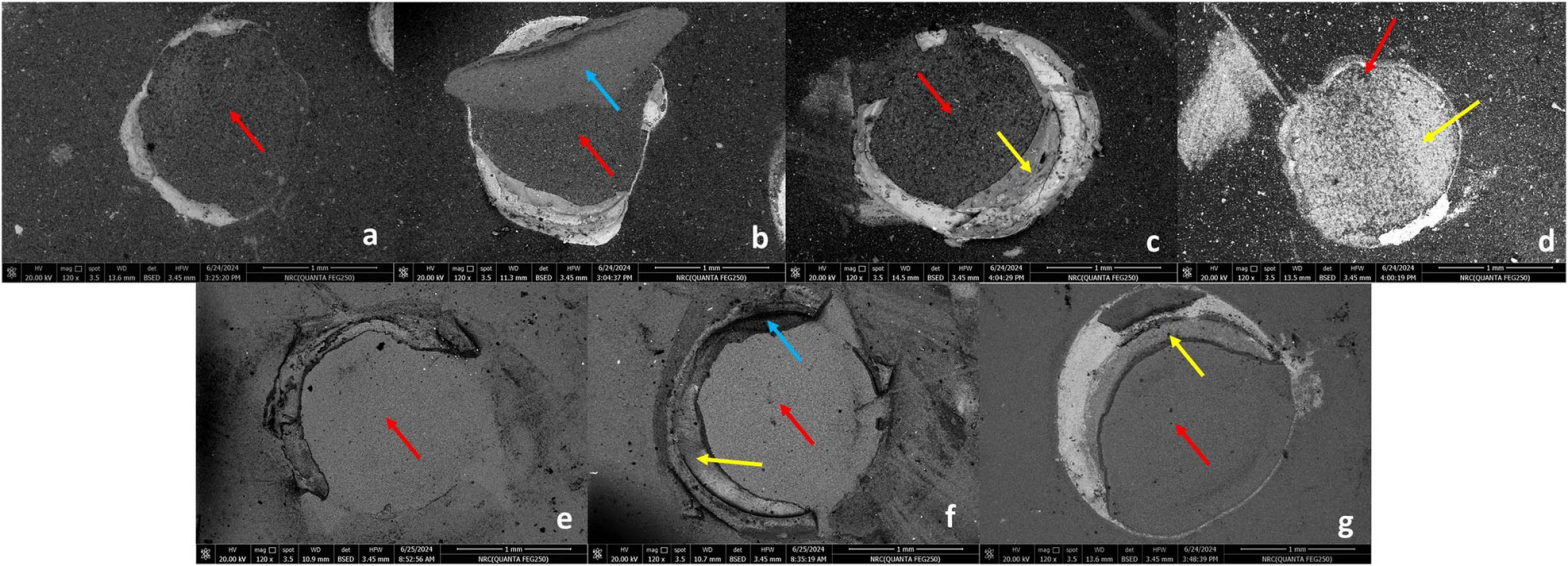
The failure modes. The adhesive (**a**, **e**) and mixed (**b**: adhesive/cohesive failure within the substrate, **c**, **g**: predominantly adhesive, **d**: predominantly cohesive within the luting agent, **f**: adhesive/cohesive failure within the substrate and the luting agent) failures observed in the present study shown by SEM at 120× magnification. The red arrows correspond to the adhesive failures, the blue arrows correspond to the cohesive failures within the substrates, the yellow arrows correspond to the cohesive failures within the luting agents. [Note: **a**, **b**, **c**, **d** are specimens of the VE tested groups, while, **e**, **f**, **g** are specimens of the GR tested groups].

No pretesting failures occurred during the preparation of the specimens or the μ-SBS testing procedures. Mean and standard deviation (MPa) of the μ-SBS values showed statistically insignificant difference among the tested groups as shown in Table 2. The failure modes revealed both adhesive and mixed failures in all groups (Fig. 3). The majority of mixed failures in all groups were predominantly adhesive (>50% of the bonded surface showed adhesive failure). However, adhesive/cohesive failure within the substrate was seen in two specimens in Group VE-F and one specimen in Group GR-F, predominantly cohesive failure within the luting agent was seen in one specimen in Group VE-C, and adhesive/cohesive failure within the substrate and the luting agent was seen in two specimens in Group GR-C (Fig. 4).

**Table 2:** Results of micro-shear bond strength test (MPa)

Micro-shear bond strength values (Mean ± SD)				
	Resin cement	Flowable composite	*P* value	Mean difference (95%CI; lower to upper)
Vita Enamic	Group (VE-C)16.83 ± 3.67	Group (VE-F) 16.59 ± 4.5	0.873	0.23 (−2.73 to 3.20)
Voco Grandio	Group (GR-C) 14.8 ± 3.6	Group (GR-F) 17.1 ± 4.36	0.114	2.30 (−5.19 to 0.59)
*P* value	0.125	0.749	----	----
Mean difference (95%CI; lower to upper)	2.03 (−0.60 to 4.65)	0.51 (−3.71 to 2.69)	----	----

*SD* standard deviation, *P* value probability level is significant at *P* ≤ 0.05, *CI* confidence interval.

## Discussion

The first and second null hypotheses were accepted, where there was statistically insignificant difference among the tested groups.

The use of hybrid ceramics has increased recently to make use of their benefits; such as high resilience, shock-absorbing properties, high milling efficiency with less marginal chipping and good polishability [5, 6].

Although resin cements were the gold standard in cementing these materials, flowable composite has been tested as valid alternative. Flowable composite possessed low viscosity with easy pre-polymerization excess material removal [21]. In addition, it’s light-cuing nature allowed for good control of the working time [21]. Although resin cements possess high flow, colour stability [14], availability in wide range of shades with possible application of try in pastes for better reproduction and visualization of the final shade [24], its low filler content still represents a challenge [14]. The increasing interest in using flowable composites for adhesive luting is to benefit from their physical properties; being more filler-loaded than resin cements, and their improved cost benefits compared to resin cements [20].

Upon comparing the shear bond strength of flowable composite and dual-cured resin cement when being used to cement feldspathic porcelain to bovine enamel, Barceleiro et al. [21], found an insignificant difference between them with the flowable composite showing insignificantly higher values. Also, Mutlu et al. [18] found that total etch flowable composites showed higher shear bond strength values after thermocycling when compared to total etch dual-cured resin cements, upon being used to cement lithium disilicate glass-ceramics to human dentin.

However, the bonding behaviour of flowable composite when luting hybrid ceramics was scarce. Thus, the present study was conducted aiming to compare the luting efficiency of flowable composite and light-cured resin cement to two advanced hybrid ceramics.

Bonding tests are commonly used in researches to evaluate the performance of adhesive systems and techniques [34]. The stronger the bond between the tooth and the restoration, the better it will resist the functional stresses [34]. Although, shear bond strength test is commonly used [34], micro-shear test was employed in the present study because it was believed to be more accurate since it tested specimens with small surface area allowing better stress distribution [34]. It was also preferred over micro-tensile bond strength test because specimen preparation in such test possessed some difficulties, required skill and the tensile forces applied are known to cause micro-cracks formation and propagation in the early stage of the test, increasing pre-test failure incidences [6, 31, 34]. However, micro-shear bond strength values can be affected by specimen geometry and loading configurations [34]; thus, standardization of all micro-cylinders’ length, diameter and testing procedures was employed in the present study.

To perform a successful micro-shear bond strength test with greater sensitivity and more homogeneous data, the bonding area should not exceed 2 mm^2^ [44, 46, 47], hence, the diameter of the micro-cylinders employed in the present study was 1.44 mm, with a total bonding area of ≈1.63 mm^2^. The employed micro-cylinders’ diameter lies within the range used in several previous studies, which utilized 1.4 mm [44, 48] and 1.5 mm [46, 47, 49] diameters. Additionally, the utilized micro-tubes internal diameter allowed easier application of the luting agent compared to narrow micro-tubes with diameters smaller than 1 mm [50].

Water storage was also performed as it is considered one of the simplest methods of aging used to simulate intra-oral conditions, with a dramatic effect on bonding [28, 51, 52]. The number of storage days varied greatly among researches testing bonding to ceramics; ranging from 0.16 up to 730 days [51]. Although some researchers performed their micro-shear bonding tests only after 24 h (1 day) of water storage; such as Yu and Wang [53], Andermatt and Özcan [40], Saglam et al. [54], and Dos Santos et al. [37], our test was performed after 21 days of water storage to simulate short-term aging. This came in agreement with Samimi et al. [51], who used two weeks aging period in deionized water prior to testing micro-shear bond strength. They believed that although short-term water storage might be considered a limitation, the small size of the bonding area might allow faster aging effect [51].

Since aging is well-known to drastically decrease the bond strength values compared to baseline [29], the present study was more concerned with comparing the bonding effect of the luting agents to two different substrates; testing two main variables, rather than comparing the aging times or protocols.

Stereomicroscope, a non-invasive magnifying instrument that allowed specimens inspection with better details than naked eyes [55], was used to detect failure modes. Scanning electron microscope was also used to help better visualization of the microstructure topography of the failed specimens at high resolution and considerable field depth [56].

Our results showed that there was statistically insignificant difference between the μ-SBS of the light-cured resin cement and the flowable composite tested, which might indicate the reliability of flowable composite as an alternative to the resin cement in terms of bonding. However, the difference in µSBS values among the tested groups might be due to the different nature of the substrates and their interaction with the luting materials, which indicated that the effect of luting agent depends on the ceramic material used [33].

In the current study, VE-C had higher µSBS than GR-C, which came in agreement with Günal-Abduljalil et al. [6], who found that VE had higher µSBS to dual-cured resin cement after air abrasion and silane application compared to GR. The present results also showed that VE-C had higher µSBS than VE-F, which also agreed with Grangeiro et al. [33], who found that µSBS of resin cement to VE was significantly higher than flowable composite with or without aging. However, Dikici and Say [22], found that the flowable composite showed statistically significantly higher micro-tensile bond strength to VE compared to dual-cured resin cement. The difference in their results might be due to using different testing protocol and the different nature of the luting cement.

All µSBS results seen in the present study were higher than the minimum acceptable bond strength values for resin materials to ceramics presented in the literature (10–12 MPa) [57]. This might be due to employing the surface treatments recommended by the manufacturer for each substrate, which enhanced their bondability. It might also be due to the fact that both the flowable composite and the resin cement had  high flow, which enhanced their penetration in the surface irregularities of the pretreated ceramic materials [33]. This result implicates the reliability of both luting agents in terms of bondability to VE and GR.

The failures seen in the present study were adhesive and mixed in nature, with the majority of the mixed failures being predominantly adhesive. This might be attributed to the low organic content present in both tested substrates; VE and GR, which might have decreased the capacity for chemical copolymerization of free monomers with the luting agent’s monomers [43].

Our results partially agreed with Beyabanaki et al. [43], who found that VE showed higher mixed failure than adhesive failures when testing their µSBS to dual-cured resin cement. However, they also found cohesive failures, which was higher than adhesive failure yet lower than mixed failure. The difference in their results might be attributed to the different nature of the luting material; being dual-cured in their study, and the different aging procedures, where they used thermocycling.

However, the present results disagreed with Günal-Abduljalil et al. [6], who found that both VE and GR had higher adhesive failures than cohesive and mixed failures when testing their µSBS to dual-cured resin cement after air abrasion and silanization, which could be attributed to the difference in the luting agent nature and aging protocol; where they applied water storage for only 24 h.

Although in vitro studies are valuable methods to evaluate dental materials’ behaviour and properties in an easy and rapid manner [28], they still lack exact simulation of the oral environment conditions. This can be considered a limitation of the present study, which also lacked the simulation of the effect of saliva, cyclic loading and thermal fluctuations. Short-term water storage aging might also be considered a limitation. Hence, further studies are recommended to compare the effect of different storage media, time and temperature on the bond strength of the tested materials, in addition to assessing the colour stability and fracture resistance of variety of ceramic materials cemented using resin cements and flowable composites.

## Conclusions

Within the limitations of this in vitro study, it can be concluded that,

- Both flowable composite and light-cured resin cement provided high bond strength when cementing VE and GR after short-term aging.
- The nature of ceramic material affects its micro-shear bond strength to luting agents.

## Data Availability

All data included in this study are available from the corresponding author upon request.
